# The Association between Endometriosis and Chronic Endometritis

**DOI:** 10.1371/journal.pone.0088354

**Published:** 2014-02-18

**Authors:** Akie Takebayashi, Fuminori Kimura, Yohei Kishi, Mitsuaki Ishida, Akimasa Takahashi, Akiyoshi Yamanaka, Kentaro Takahashi, Hiroshi Suginami, Takashi Murakami

**Affiliations:** 1 Department of Obstetrics and Gynecology, Shiga University of Medical Science, Seta Tsukinowa-cho, Otsu, Shiga, Japan; 2 Department of Obstetrics and Gynecology, Takanohara Central Hospital, Nara-shi, Nara, Japan; 3 Department of Clinical Laboratory Medicine and Division of Diagnostic Pathology, Shiga University of Medical Science, Seta Tsukinowa-cho, Otsu, Shiga, Japan; 4 Department of Community Perinatal Medicine, Shiga University of Medical Science, Seta Tsukinowa-cho, Otsu, Shiga, Japan; University of Edinburgh, United Kingdom

## Abstract

**Objective:**

To evaluate the association between endometriosis and chronic endometritis.

**Methods:**

Endometrial specimens were obtained from 71 patients, 34 with endometriosis (endometriosis group) and 37 without endometriosis (non-endometriosis group), who underwent hysterectomy, and the specimens were immunostained for the plasmacyte marker CD138. The rate of chronic endometritis was compared between the endometriosis group and the non-endometriosis group. Furthermore, the 71 patients were also divided into two groups, 28 with chronic endometritis (chronic endometritis group) and 43 without chronic endometritis (non-chronic endometritis group). Logistic regression analysis was performed with variables including age, body mass index (BMI), gravidity and parity, and diagnoses of leiomyoma, adenomyosis, and endometriosis on pathology to examine the independent effect of each variable on chronic endometritis. Patients suffering from cervical invasive carcinoma, endometrial carcinoma, and endometrial polyps or treated with gonadotropin-releasing hormone agonists, progestins, or oral contraceptives before surgery were excluded.

**Results:**

Chronic endometritis was identified in 52.94% of the endometriosis group and 27.02% of the non-endometriosis group (p<0.05). Logistic regression analysis revealed that endometriosis was associated with chronic endometritis.

**Conclusions:**

This result suggests a strong association between endometriosis and chronic endometritis.

## Introduction

Chronic endometritis is a persistent inflammation of uterine endometrium, and it is diagnosed histopathologically as plasmacyte infiltration within the endometrial stromal compartment [1], [2]. It is usually asymptomatic or presents only with subtle symptoms such as abnormal uterine bleeding, pelvic pain, dyspareunia, and leucorrhea. It has been thought not to affect the reproductive status and ability of affected women [1]. However, recent studies have reported that chronic endometritis is associated with infertility and recurrent abortion; it has been identified in 12–46% of infertile patients, 30% of repeated implantation failures after in vitro fertilization-embryo transfer, 28% of unexplained infertility, and 12% of unexplained recurrent miscarriages [3]–[7]. In chronic endometritis, a number of immune cells including plasma cells is found in the endometrium. Thus, given the presence of abnormal immune cells in the endometrium, chronic endometritis may affect the development and maintenance of other reproductive diseases.

Endometriosis is characterized by the occurrence of endometrial-like tissue outside the uterus. It is a chronic disease with symptoms such as dysmenorrhea, dyspareunia, severe chronic pelvic pain, and infertility that interfere severely with social life and sexual and psychological well-being, generally impairing the quality of life of affected women [8]. Endometriosis is widely documented as an inflammatory disease with an abnormal immune response. Some evidence regarding the important roles of immune cells in facilitating the development and maintenance of endometriotic lesions has been reported [9]–[11]. The distribution of immune cells in the pelvic cavity has been reported to differ between endometriosis patients and non-endometriosis patients [12]–[19]. Increased activation of macrophages, along with increased secretion and synthesis of different pro-inflammatory mediators, cytokines, tumor necrosis factor-α, interleukins, RANTES, platelet activating factors, fibroblast growth factors, hepatocyte growth factor (HGF), macrophage-derived growth factor, vascular endothelial growth factor, angiogenesis factor, and fibronectin has been reported at ectopic sites in women with endometriosis. Their effects may facilitate the development and maintenance of endometriosis [13], [15], [16], [20], [21]. Changes in the immune response within the eutopic endometrium of women with endometriosis have also been investigated; the distribution of macrophages in eutopic endometrium differs between endometriosis patients and non-endometriosis patients [21], [22]. This suggests that endometriosis is related to abnormal immune systems in the eutopic endometrium. Moreover, compared with eutopic endometrium in non-endometriosis patients, gene expressions and protein secretions of eutopic endometrium are known to be modified in endometriosis patients [23]–[25]. Thus, the immune system appears to be significantly modified not only at the endometriotic site, but also within the eutopic endometrium of women with endometriosis, affecting the viability and function of the eutopic endometrium.

It is therefore possible to hypothesize that endometriosis may be related to chronic endometritis. To the best of our knowledge, there have been no reports investigating this relationship. The aim of this study was to clarify the hypothesis by comparing the incidence of chronic endometritis between endometriosis and non-endometriosis patients. The present results demonstrated an association between endometriosis and chronic endometritis.

## Materials and Methods

### Materials

A total of 71 untreated patients, 34 patients with endometriosis (endometriosis group) and 37 without endometriosis (non-endometriosis group), having normal menstrual cycles who underwent hysterectomy for gynecological disease at Shiga University of Medical Science and Takanohara Central Hospital from April 2001 to December 2012 was enrolled. This study conformed to the Clinical Research Guidelines of Shiga University of Medical Science and Takanohara Central Hospital, and was approved by the research ethics committees of both establishments. Written, informed consent to participate in this study was obtained from all patients.

### Immunohistochemistry

Paraffin-embedded endometrial specimens were used for the study. All samples were obtained by hysterectomy. The investigators were blinded at this stage to the clinical parameters of the specimens. The specimens were cut into 4-µm-thick sections, placed onto a 42°C water bath, and mounted onto 3-aminopropyltriethoxysaline-coated slides (FRC-05; Matsunami, Osaka, Japan). The sections were confirmed to include a sufficient endometrial area for the analysis. After deparaffinization and rehydration, the slides were placed in pre-heated tris-ethylenediaminetetraacetic acid (EDTA) (pH 9.0) and boiled at 98°C for 10 minutes. They were then immersed in 3% hydrogen peroxide for 10 minutes to block endogenous peroxidase. After washing, the sections were covered with blocking solution (PK-4002; VECTASTAIN, Vector Laboratories, Burlingame, CA) for 20 minutes to suppress non-specific antibody binding. The sections were immunostained with anti-CD138 antibody (B-A38; Nichirei Corp., Tokyo, Japan) for 1 hour at room temperature, followed by washing in phosphate-buffered saline (PBS) and incubation with horseradish peroxidase-conjugated secondary antibody (MAX-PO; Nichirei Corp.) for 30 minutes at room temperature, and developed with diaminobenzidine (VECTOR SK-4100; VECTASTAIN, Vector Laboratories). The slides were counterstained with Mayer's hematoxylin solution, followed by dehydration and mounting.

### Analysis

In this study, a sufficient endometrial stromal area of at least two large sections was evaluated in each patient, and the number of immunoreactive cells was counted under a light microscope (400-fold magnification, a high powered field; HPF). The density of immunoreactive cells was enumerated in 10 non-overlapping random stromal areas, and chronic endometritis was diagnosed when one or more plasma cells were detected, since Kasius et al. reported that the presence of only one plasma cell in the endometrial stromal area was sufficient to diagnosis chronic endometritis. The diagnosis of chronic endometritis was performed by two separate blinded investigators.

After all procedures to diagnose chronic endometritis were completed, the clinical parameters were disclosed. Patients treated with gonadotropin-releasing hormone agonist, progestins, or oral contraceptives before surgery were excluded, as were specimens obtained during menstruation. Specimens showing cervical invasive carcinoma, endometrial carcinoma, and endometrial polyps were excluded. The clinical characteristics were obtained from the documents related to the preoperative assessment. The presence of endometriosis was identified by the operation records and the video documentation in the cases treated by laparoscopic surgery. The endometriosis stage was scored by the revised staging system of the American Society of Reproductive Medicine [26]. Using the standard criteria, histological analysis revealed the menstrual cycle date of the specimens [27].

Statistical analysis was performed using Graph Pad Prism 5 (GraphPad Software Inc., La Jolla, CA) and SPSS version 20 (SPSS Inc, Chicago, IL). Each dataset was analyzed for a normal distribution using the Kolmogorov-Smirnov test, and Student's *t*-test or the non-parametric Mann-Whitney U test was used depending on the distribution pattern. The statistical significance of the difference in the rates of chronic endometritis between the endometriosis group and the non-endometriosis group was examined using chi-square analysis.

Significant differences were observed between the endometriosis and the non-endometriosis groups in some aspects of the clinical characteristics and pathological diagnoses. Therefore, the 71 patients were divided into two groups, 28 patients with chronic endometritis (chronic endometritis group) and 43 without chronic endometritis (non-chronic endometritis group), and stepwise logistic regression analysis was performed to examine the independent effect of each variable on chronic endometritis. The analyzed variables included age, BMI, gravidity and parity, and the pathological diagnoses of leiomyoma, adenomyosis, and endometriosis.

After analyzing the relationship between endometriosis and chronic endometritis, the rate of chronic endometritis was also analyzed at each rASRM stage of endometriosis to clarify the impact of the disease progression of endometriosis on chronic endometritis. Furthermore, to clarify the effect of endometrioma of the ovary and deep recto-vaginal pouch endometriosis on the incidence of chronic endometritis, the endometriosis group was divided into those with and without such lesions, and the rates of chronic endometritis were calculated.

## Results

### Clinical parameters in the endometriosis group and the non-endometriosis group

There were 34 patients in the endometriosis group and 37 in the non-endometriosis group. Patients' background clinical characteristics are shown in Table 1. No differences were observed between the endometriosis group and the non-endometriosis group in age, BMI, menstrual phase (the ratio of proliferative phase to secretory phase), menstrual duration, and frequency of dysmenorrhea. Parity was significantly higher (p = 0.0493) in the non-endometriosis than in the endometriosis group, while gravidity was not significantly different. The endometriosis group included significantly more specimens with adenomyosis (p<0.001) and fewer specimens with leiomyoma (p = 0.003). The number of specimens including carcinoma in situ (CIS) was not different. There were 9 patients in the endometriosis group and two in the non-endometriosis group with multiple pathological diagnoses.

**Table 1:** Clinical characteristics and pathological diagnoses of uterus (endometriosis and non-endometriosis groups).

	Endometriosis group (n = 34)	Non Endometriosis group (n = 37)	P value
**Age (years)**	44.15±3.65	43.15±2.75	0.711
**BMI (kg/m^2^)**	22.08±4.83	21.60±3.14	0.940
**Gravidity**	1.94±1.43	2.43±1.52	0.211
**Parity**	1.38±1.04	1.92±0.95	0.049*
**Menstrual average cycle (days)**	27.50±2.32	27.72±2.58	0.664
**Menstrual duration (days)**	6.73±1.64	6.62±2.41	0.272
**Dysmenorrhea (%)**	58.82 (20/34)	43.24 (16/37)	0.119
**Proliferative phase (%)**	55.88 (19/34)	54.05 (20/37)	0.877
**Secretory phase (%)**	44.12 (15/34)	45.95 (17/37)	0.877
**Leiomyoma (%)**	67.65 (23/34)	94.59 (35/37)	0.003*
**Adenomyosis (%)**	47.06 (16/34)	8.11 (3/37)	<0.001*
**CIS (%)**	2.94 (1/34)	5.41(2/37)	0.532

*Significant difference at p<0.05.

There are 9 patients in the endometriosis group and 2 in the non endometriosis group with multiple pathological diagnoses of uterus.

### Rate of chronic endometritis in the endometriosis and non-endometriosis groups

**Figure 1:**
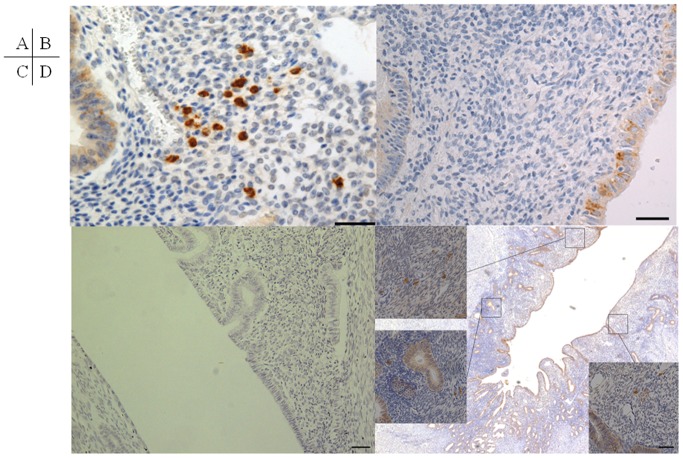
Immunohistochemistry for CD138. (A) Plasma cells immunostained by CD138 are detected in the stromal compartment of chronic endometritis. (B) Plasma cells are not detected in the stromal compartment of non-chronic endometritis, although CD138 is also immunostained in the epithelium of all sections. (C) No immunostaining is detected in the negative control in which anti-CD138 antibody has been omitted. (D) Plasma cells can be found in at least three of ten non-overlapped random fields once a plasma cell is detected in a stromal compartment. Since chronic endometritis does not localize focally but expands to involve the entire endometrial stroma, a number of plasma cells is detected in random endometrial stromal compartments in chronic endometritis. Bar = 100 µm.

Chronic endometritis was identified by punctate immunostaining for CD138 in the stromal compartment (Figure 1A). CD138 was detected in epithelia of all sections including non-chronic endometritis (Figure 1 B), while no immunostaining was detected in the negative control in which anti-CD138 antibody was omitted (Figure 1C). In the patients in whom a plasma cell was detected in a stromal compartment, multiple plasma cells were found in other endometrial stromal areas. Plasma cells could be found in at least three of ten non-overlapped random fields once a plasma cell was detected in a stromal compartment. In the patients in whom no plasma cell was detected in 10 non-overlapped random fields of endometrial stromal area, no plasma cells were detected in the whole endometrial stromal area of the sections. Thus, chronic endometritis was defined as the presence of multiple plasma cells in 10 non-overlapped random fields of endometrial stromal area (Figure 1D). Chronic endometritis was detected in 52.94% of the endometriosis group and 27.02% of the non-endometriosis group; the rate was significantly higher in the endometriosis group than in the non-endometriosis group (p = 0.0311) (Table 2). Since there were also significant differences in terms of adenomyosis, leiomyoma, and parity between the endometriosis and non-endometriosis groups (Table 1), it is possible that these factors contributed to the result. Therefore, the effects of endometriosis, adenomyosis, leiomyoma, and other background factors on chronic endometritis were investigated by stepwise logistic regression analysis.

**Table 2:** Rate of chronic endometritis in the endometriosis and non-endometriosis groups.

	Endometriosis group (n = 34)	Non Endometriosis group (n = 37)	P value
**Positive rate (%)**	52.94 (18/34)	27.02 (10/37)	0.031*

### Stepwise logistic regression analysis

The 71 patients with or without endometriosis were divided into two groups, 28 patients with chronic endometritis (chronic endometritis group) and 43 patients without chronic endometritis (non-chronic endometritis group). Their background characteristics and pathological results are listed in Table 3. Logistic regression analysis was performed with variables including age, BMI, gravidity and parity, and the diagnoses of leiomyoma, adenomyosis, and endometriosis on pathology. No relationships were found for age, BMI, gravidity and parity, adenomyosis, leiomyoma, and CIS. Only the variable “endometriosis” was found to be related to chronic endometritis (Table 4).

**Table 3:** Clinical characteristics and pathological diagnoses of uterus (chronic endometritis and non-chronic endometritis groups).

	Chronic Endometritis group (n = 28)	Non Chronic Endometritis group (n = 43)	P value
**Age (years)**	44.00±3.43	44.33±3.06	0.781
**BMI (kg/m^2^)**	22.93±4.76	21.94±3.45	0.537
**Gravidity**	1.93±1.27	2.37±1.60	0.233
**Parity**	1.54±1.00	1.74±1.05	0.384
**Menstrual average cycle (days)**	27.44±2.39	27.72±2.51	0.679
**Menstrual duration (days)**	6.81±1.49	6.57±2.41	0.135
**Dysmenorrhea (%)**	57.14 (16/28)	48.84 (21/43)	0.494
**Diagnosis of endometriosis (%)**	64.29 (18/28)	37.21 (16/43)	0.026*
**Proliferative phase (%)**	64.29 (18/28)	48.83 (21/43)	0.201
**Secretory phase (%)**	35.71 (10/28)	51.16 (22/43)	0.201
**Leiomyoma (%)**	75.00 (21/28)	86.04 (37/43)	0.240
**Adenomyosis (%)**	28.57 (8/28)	25.58 (11/43)	0.781
**CIS (%)**	3.57 (1/28)	4.65 (2/43)	0.658

*Significant difference at p<0.05.

There are 3 patients in the chronic endometritis group and 9 in the non chronic endometritis group with multiple pathological diagnoses of uterus.

**Table 4:** Logistic regression analysis for chronic endometritis.

Variable	βvalue	P value	Odds ratio	95% CI
**Endometriosis**	1.111	0.028	3.037	1.129–8.174

### Chronic endometritis at each stage of endometriosis

The rate of chronic endometritis was analyzed at each stage of endometriosis. Chronic endometritis was found in 40.0% of stage I endometriosis, 50.0% of stage II, 70.0% of stage III, and 46.7% of stage IV (Table 5). No significant difference was found between early stages (I+II) and late stages (III+IV) (Table 6). The effects of endometrioma of the ovary and deep recto-vaginal pouch endometriosis on the incidence of chronic endometritis were also evaluated. Chronic endometritis was found in 52.2% (12/23) of endometriosis with ovarian endometrioma and 54.5% (6/11) of endometriosis without ovarian endometrioma; no significant difference was seen between endometriosis with and without ovarian endometrioma. Chronic endometritis was found in 33.3% (1/3) of endometriosis with and 54.8% (17/31) of endometriosis without deep recto-vaginal pouch endometriosis; no significant difference was found between them.

**Table 5:** Rates of chronic endometritis in each stage of endometriosis and comparison between early stages (I+II) and late stages (III+IV).

r-AFS	Positive rate (%)
**Stage I**	40.00 (2/5)
**Stage II**	50.00 (2/4)
**Stage III**	70.00 (7/10)
**Stage IV**	46.67 (7/15)

**Table 6:** Comparison of the rate of chronic endometritis between early stages (I+II) and late stages (III+IV).

	Stage I+II	Stage III+IV	p-value
**Positive rate (%)**	44.44 (4/9)	56.00 (14/25)	0.7027

## Discussion

Chronic endometritis is generally an asymptomatic condition and, therefore, difficult to diagnose. Although pathological endometrial alteration has been described as indicative of chronic endometritis, the diagnosis ultimately relies on the presence of plasma cells on histological examination [1], [2]. To make it clear, immunostaining for detecting plasma cells has been recommended. Bayer-Garner and Korourian reported that the recognition of plasma cells is difficult in endometrial tissue that includes a prominent spindle cell stromal component. They showed that immunohistochemistry for the transmembrane heparan sulfate proteoglycan syndecan-1 (CD138) improved the diagnostic accuracy of chronic endometritis compared with morphological analysis without immunohistochemistry [28]. Thus, immunostaining for CD138 was used to detect plasma cells in the specimens in the present study.

Although endometrial biopsy samples have been used for the diagnosis of chronic endometriosis, these specimens may not be sufficient to detect stromal plasmacyte infiltrates, since examination of the samples cannot detect the plasmacytes near and/or within the endometrial basal layer [29]. The other concern with using biopsy samples is that plasma cells originating in epithelium may invade into the stromal compartment during the process of curettage. Especially in the proliferative phase, the epithelial and glandular area is relatively larger, and the curettage may destroy tissue structure, causing plasma cells to lie in the stromal compartment. Moreover, a sufficient endometrial specimen cannot be obtained from patients with thinner endometrium. For these reasons, specimens obtained by endometrial biopsy were avoided in the present study. However, the use of hysterectomy samples rather than endometrial biopsy samples was a limitation of the study.

In the present study, the incidence of chronic endometritis was significantly higher in the endometriosis group than in the non-endometriosis group. Ideally, to study the relationship between endometriosis and chronic endometritis, only the normal uterus with myometrium and endometrium should be examined. However, all of the specimens examined in this study included some lesions in the myometrium, and the pathological background was different between the endometriosis and non-endometriosis groups. Therefore, the specimens were divided into the chronic endometritis group and the non-chronic endometritis group, and stepwise logistic regression analysis with the variables related to background characteristics and pathological aspects was performed to examine the independent effect of each variable on chronic endometritis. Only the variable “endometriosis” was found to be related to chronic endometritis. In other words, it was found that only endometriosis was a significant predictor of chronic endometritis. This result suggests a strong association between chronic endometritis and endometriosis.

Although previous reports defined chronic endometritis as plasmacyte infiltration within the endometrial stromal compartment, no studies have defined the number of infiltrated plasmacytes and the areas to count. Kasius et al. stated that the presence of only one plasma cell is sufficient to diagnose chronic endometritis [29]. In the present study, chronic endometritis was diagnosed when there was at least one plasma cell in 10 non-overlapping random stromal areas. In patients in whom one or more plasma cells were detected in a stromal compartment, multiple plasma cells were found in other endometrial stromal areas. It was found that multiple plasma cells could be identified in at least three of ten non-overlapped random fields once a plasma cell was detected in a stromal compartment. Since chronic endometritis does not localize focally but expands to involve the entire endometrial stroma, a number of plasma cells is detected in random endometrial stromal compartments in chronic endometritis. Conversely, when no plasma cells were detected in 10 non-overlapped random fields of endometrial area, plasma cells were not detected in the entire endometrial stromal area of the sections.

**Figure 2:**
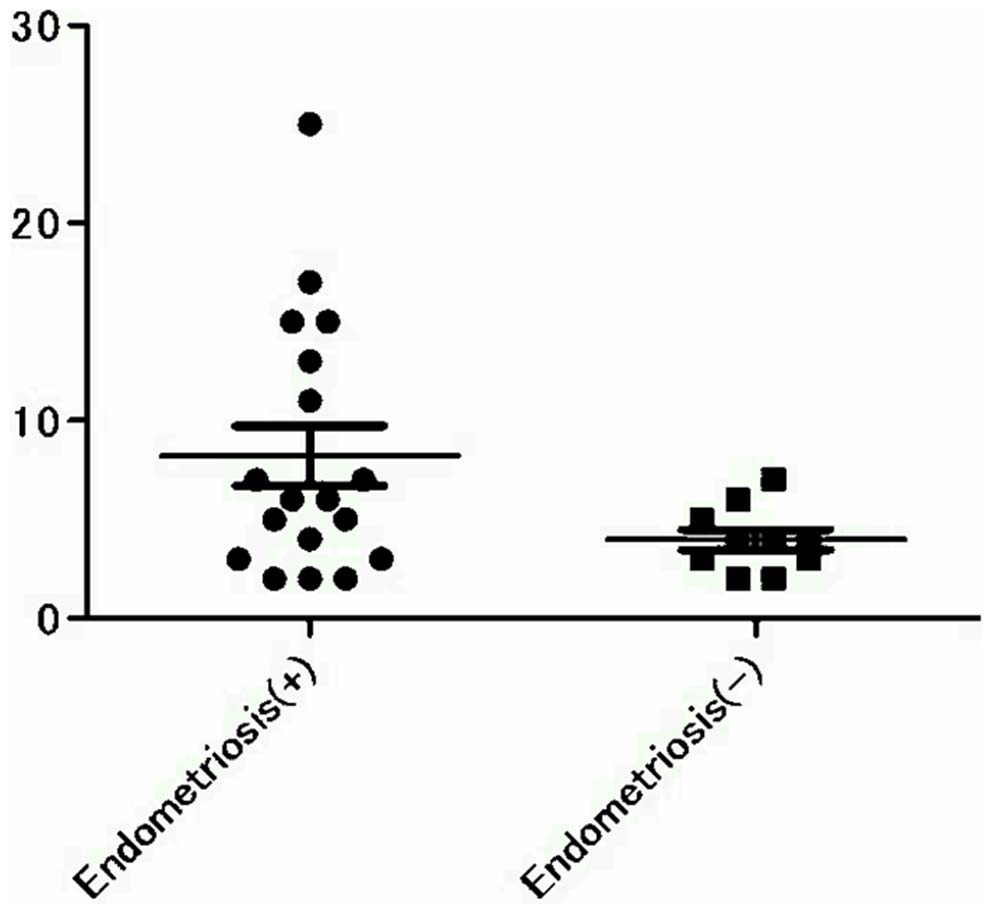
The number of accumulated immunoreactive cells in one HPF. All cases with over 11 plasma cells in one HPF belong to the endometriosis group.

Kitaya et al. immunostained 234 specimens obtained by hysterectomy due to benign gynecological disease and reported that chronic endometritis was identified in 11.1% of the endometrial specimens [30]. Compared to their result, the rate of chronic endometritis was higher in the present study. They reported that the number of immunoreactive cells in 10 non-overlapping stromal areas varied from 12 to 56 in 26 chronic endometritis cases. They excluded the patients with less than 12 immunoreactive cells. The difference in the diagnostic criteria may explain the dissimilar results. In the present study, the stromal area where the greatest number of plasma cells was present in each patient was evaluated, and the greatest number in one HPF for each patient was recorded. When comparing the number of patients with more than 6 plasma cells in one HPF, the overall percentage was significantly higher in the endometriosis group than in the non-endometriosis group (29.41% vs. 5.4%, p = 0.0101). Moreover, when the numbers of patients with over 11 plasma cells in one HPF were compared, the percentage was significantly higher in the endometriosis group than in the non-endometriosis group (17.65% vs. 0%, p = 0.0094). All patients with more than 11 plasma cells in one HPF belonged to the endometriosis group (Figure 2). Based on these results, two things may be suggested. First, even if the criterion of chronic endometritis is different, the prevalence of chronic endometritis is higher in the endometriosis group. Second, the inflammation of chronic endometritis is severe in endometriosis patients. Wood et al. claimed that increased mucosal plasma cell counts in the duodenal mucosa of patients with celiac disease mirrored increased local production of immunoglobulins [31] that increase in response to infection, chronic inflammation, and autoimmune disease [32]. Thus, when thinking about the endometrium, it is possible to say that accumulation of plasmacytes is related to the severity of chronic inflammation in the endometrium, and chronic endometritis in endometriosis may be more active, but the reason why they accumulate in chronic endometritis still needs to be identified.

The rate of chronic endometritis at each r-AFS stage was analyzed (Table 5). The rates were not correlated with stage, and deep endometriotic lesions were not related to chronic endometritis. These results suggest that chronic endometritis may be an independent complication of endometriosis or may be involved in the pathogenesis of endometriosis, since chronic endometritis appeared even in Stage I.

Since the uterine cavity is connected to the pelvic cavity by the oviducts, the cells and humoral factors can pass between the cavities. In other words, some humoral factors or substances produced by endometriosis in the pelvic cavity can return to the uterine cavity and may induce plasma cell infiltration in the entopic endometrial stromal compartment. Conversely, the abnormal plasma cells within the eutopic endometrium can flow into the pelvic cavity with shed endometrium during menstruation, and this may cause or maintain endometriosis.

The role of plasma cells in eutopic endometrium is likely to eliminate bacteria, some organelles, and nascent neoplastic cells. However, chronic endometritis may contribute to the promotion of tumor development. Approximately 25% of all cancer cases is linked to chronic inflammation caused by chronic infections (e.g. hepatitis B and C viruses, human papilloma viruses 16 and 18, and *Helicobactor pylori*), autoimmune diseases (e.g. inflammatory bowel disease), or inflammatory conditions of uncertain origin [33], [34]. Exogenous factors are able to trigger a persistent inflammatory response. Furthermore, an inflammatory component is present in the microenvironment of most tumors that are not epidemiologically related to inflammation [33]. Although endometriosis is not cancer, the development of endometriosis mimics the process of metastasis in cancer. Several studies have highlighted that immune cell plasticity is co-opted by tumors to their own advantage. In early phases, high production of inflammatory mediators (e.g., IL-12,TNF, and reactive oxygen species) activates an adaptive immune response capable of eliminating nascent neoplastic cells, but also appears to support neoplastic transformation [35], [36]. Inside the uterine cavity, chronic endometritis may contribute to transform normal eutopic endometrium to endometriotic tissue that can invade the pelvic cavity.

In summary, this study revealed that endometriosis is associated with chronic endometritis. However, the cause of chronic endometritis in endometriosis remains unclear; it may be an infection or the endometriosis itself. Further studies are needed in this area.
